# Changes of medium-latency SEP-components following peripheral nerve lesion

**DOI:** 10.1186/1749-7221-1-4

**Published:** 2006-10-20

**Authors:** Ruediger Stendel, Uwe Jahnke, Max Straschill

**Affiliations:** 1 Department of Neurosurgery, Charité – Universitätsmedizin Berlin, Campus Benjamin Franklin, Berlin, Germany; 2 Department of Neurology and Clinical Neurophysiology, Fachklinik Neustadt, Neustadt, Germany; 3 Department of Clinical Neurophysiology, Charité – Universitätsmedizin Berlin, Campus Benjamin Franklin, Berlin, Germany

## Abstract

**Background:**

Animal studies have demonstrated complex cortical reorganization following peripheral nerve lesion. Central projection fields of intact nerves supplying skin areas which border denervated skin, extended into the deafferentiated cortical representation area. As a consequence of nerve lesions and subsequent reorganization an increase of the somatosensory evoked potentials (SEPs) was observed in cats when intact neighbouring nerves were stimulated. An increase of SEP-components of patients with nerve lesions may indicate a similar process of posttraumatic plastic cortical reorganization.

**Methods:**

To test if a similar process of post-traumatic plastic cortical reorganization does occur in humans, the SEP of intact neighbouring hand nerves were recorded in 29 patients with hand nerve lesions. To hypothetically explain the observed changes of SEP-components, SEP recording following paired stimulation of the median nerve was performed in 12 healthy subjects.

**Results:**

Surprisingly 16 of the 29 patients (55.2%) showed a reduction or elimination of N35, P45 and N60. Patients with lesions of two nerves showed more SEP-changes than patients with a single nerve lesion (85.7%; 6/7 nerves; vs. 34.2%; 13/38 nerves; Fisher's exact test, p < 0.05). With paired stimulation a suppression of the amplitude of N20, P25 and P45 (p < 0.05; sign test), and a marked increment of N35 (p < 0.05; sign test) and N60 (not significant; sign test) of the second response could be observed.

**Conclusion:**

The results of the present investigation do not provide evidence of collateral innervation of peripherally denervated cortical neurons by neurons of adjacent cortical representation areas. They rather suggest that secondary components of the excitatory response to nerve stimulation are lost in cortical areas, which surround the denervated region.

## Background

Animal studies have shown complex reorganization of the somatosensory cortex following lesions of peripheral sensory nerves. About two months after nerve lesion, a response could be found of the initially deafferentiated cortical neurons to stimulation of adjacent skin areas innervated by other nerves. The initially deafferentiated cortical area was occupied by new afferents from adjacent nerves [1-8]. In all of these nerve lesion studies, the cortical neuron population supplied by intact nerves increased by the proportion of the re-innervated deafferentiated neurons. Since the somatosensory evoked potential (SEP) as the sum of the cortical neuronal reactions to electric stimulation of a peripheral nerve increases in amplitude with the number of activated cortical elements, one would expect an increase in the amplitude of the SEP to occur upon stimulation of intact adjacent hand nerves. Such an increase has indeed been observed in the cat under such conditions [4]. After peripheral nerve lesion, a persistent increase in the amplitude of the P2-component upon stimulation of paw areas which are adjacent to the peripherally denervated skin areas could be observed [4].

Are the SEP-components of patients with hand nerve lesions likewise increased, when the other intact hand nerves are electrically stimulated? The present study was performed to provide an answer to this question and to obtain evidence of plastic reorganization of the somatosensory cortex following hand nerve lesions in humans.

## Patients and Methods

A total of 29 patients (45 investigated nerves) (17 m, 12 f; mean age 36.5 ± 3.7 years) were included in this study. The patients were subdivided into four groups according to the extent of the nerve damage and the consecutive persisting sensory deficits (Table 1). The subjects had suffered injuries of one or two nerves (median nerve, radial nerve, ulnar nerve, superficial ramus of radial nerve, and dorsal ramus of ulnar nerve) in the region of the wrist or forearm 3 months to 8 years prior to the study with persisting sensory deficits in the area supplied by the damaged nerves. The nerve lesion had been treated conservatively or surgically. Patients with polyneuropathy, degenerative neurological disorders, status post plastic surgery with skin transplantation in the area of the arms and hands, alcohol or drug abuse, and status post chemotherapy were excluded.

**Table 1:** Pathological medium-latency SEP-components. Proportion of pathological medium-latency SEP-components in 29 patients with lesions of neighbouring hand nerves in relation to extent of nerve lesion.

	1 Nerve affected n (%)			2 Nerves affected n (%)		
	Hypaesthesia	Anaesthesia	Total	Hypaesthesia	Anaesthesia	Total
**Number of patients**	18	5	23	3	3	6
**Investigated nerves**	27	11	38	4	3	7
**Pathological SEP-components**	8 (**29.6**)	5 (**45.5**)	13 (**34.2**)*	3 (**75**)	3 (**100**)	6 (**85.7**)*

* p < 0.05

The SEPs of the hand nerves supplying areas adjacent to peripherally denervated skin areas were evoked electrically using rectangular impulses (0.2 msec duration; 3 impulses per second; intensity: slightly above the motor threshold for mixed nerves and thrice the threshold intensity for purely sensory nerves; average of 1000 responses) and compared with the SEPs of the same nerves in the intact contra-lateral hand. The SEPs were recorded using the device and software for data recording and analysis "Viking" (Nicolet GmbH, 63798 Kleinostheim, Germany). All measurements were performed twice and evaluated by two independent investigators. The means of both recordings were used for statistical analysis. The affected nerve or nerves were investigated to demonstrate complete or incomplete damage. Responses were recorded using sintered silver chloride bridge electrodes from the points C'3 or C'4 with the reference placed in Cz according to the international 10–20-system [9].

The latencies and amplitudes (baseline-to-peak and peak-to-peak, respectively) of the components N20, P25, N35, P45, and N60 were determined; mean values of two measurements calculated and the frequency of component-losses recorded. The SEP components were defined as pathological in terms of the study aim if they were absent or if the amplitude was less than 50% of the value on the contra-lateral side in two recordings.

To find a hypothetical explanation of the observed changes, in a second part of the study pairs of electrical pulses (intensity and amplitude as described above) at 3 interstimulus-intervals (ISI) (100, 150 or 200 msec) were applied to the median nerve of 12 healthy subjects (9 m, 2 f; mean age 31 ± 5.7 years). The amplitudes (peak-to-baseline and peak-to-peak, respectively) were determined and mean values of three measurements calculated. The mean values following first and second stimulation were compared.

All experiments were performed in agreement with the local ethics committee and after having informed consent of each patient.

## Results

**Figure 1:**
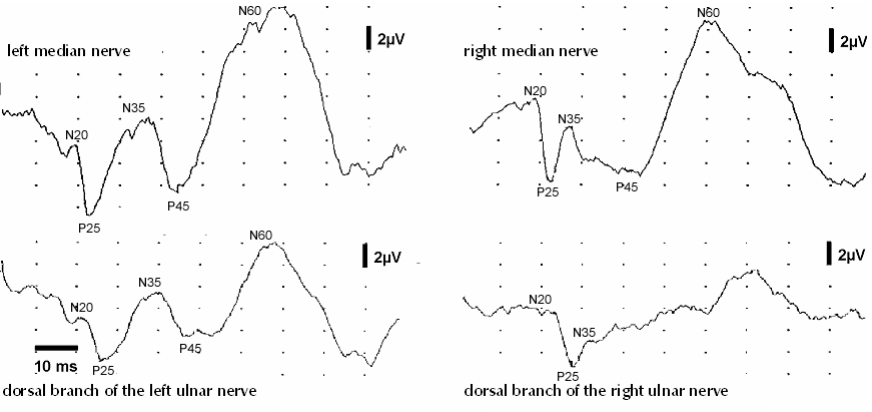
**Changes of medium-latency SEP-components following nerve lesion**. Changes of secondary SEP-components following complete right radial nerve lesion. Primary components (N20, P25) remain unaffected, while N35, P45 and N60 are depressed or abolished, when the intact directly neighbouring hand nerve is stimulated.

The primary SEP-components (N20, P25) of intact hand nerves remained unaffected by lesions of the neighbouring nerves. However, the amplitudes of the secondary, medium-latency components N35, P45 and N60 were markedly reduced or absent in 16 out of 29 patients (55.2%) or in 19 out of 45 nerves (42.2%) studied, whereas no significant differences of latencies could be found (Fisher's exact test, p > 0.05; Table 1). This amplitude change occurred only in those SEP, which were evoked from nerves with supply areas bordering directly the anaesthetic area (Figure 1).

The most frequent observed change was a depression of all 3 components, which occurred in 9 out of 29 patients (32.2%) or in 11 out of 45 nerves (24.4%) including all patients of group 4 (complete lesion of two nerves). An isolated depression of N60 or N35 was seen in 5 (17.2%), combined depression of P45 and N60 in 2 patients (6.9%). The proportion of SEP abnormalities was significantly higher in patients with lesions of two nerves as compared to patients with a single nerve lesion Patients with lesions of two nerves showed significantly more SEP-changes than those with a single nerve lesion (6/7 nerves; 85.7% vs. 13/38 nerves; 34.2%; p < 0.05, Fisher's exact test).

**Figure 2:**
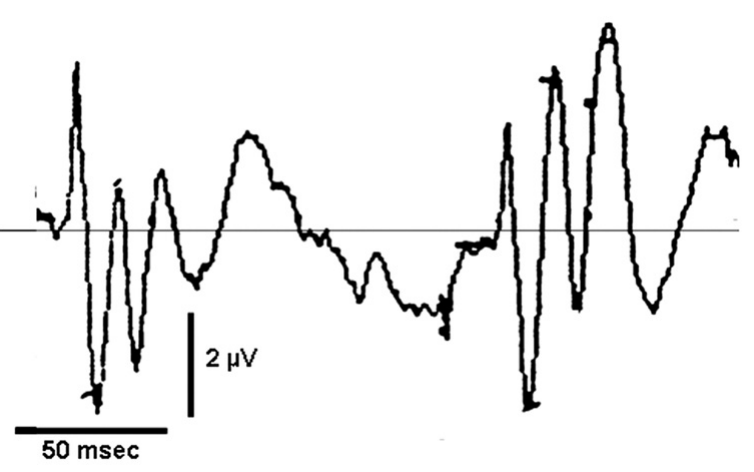
**Influence of paired stimulation on SEP-components**. Paired stimulation of the right median nerve with an inter-stimulus interval of 150 msec in a healthy subject. N20, P25 and P45 are depressed while N35 and N60 increase after the second stimulus.

**Figure 3:**
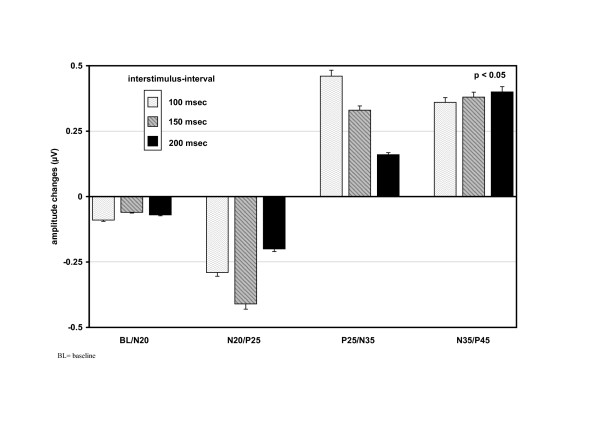
**Changes of SEP-components following paired stimulation**. Paired stimulation of median nerves of 12 healthy subjects at three different inter-stimulus intervals. The y-axis shows the changes of the SEP-components following paired stimulation. The primary SEP-components have decreased, whereas the post-primary components have increased.

With paired stimulation a suppression of N20, P25 and P45 (p < 0.05; sign test), and a marked increment of N35 (p < 0.05; sign test) of the second response could be observed (Figures 2 and 3).

## Discussion

Animal studies have demonstrated a persisting increase of ulnar evoked primary SEP-components after radial nerve lesion [4]. In contrast, the results of the present study in humans demonstrated no change of primary SEP-components of intact neighbouring hand nerves following hand nerve lesions. Surprisingly, 16 out of 29 patients (52.2%) showed a marked amplitude reduction of the medium-latency components N35, P45 and N60. The shortest interval between lesion and SEP recording with loss of components was 3 months. To find a hypothetical explanation for the observed SEP-changes, it was necessary to look into the mechanisms of SEP-component generation.

The thalamo-cortical volley in response to hand nerve stimulation evokes a primary EPSP in neurons of middle cortical layers. The human scalp correlates of this depolarizing response are the primary components N20 (area 3b) and P25 (area 1). The primary EPSP is immediately followed by a primary IPSP and after 15–20 msec by a secondary EPSP [10-12]. This secondary EPSP increases with repetitive stimulation at a rate of 6–12/sec (augmenting reaction), while the primary EPSP is depressed [13-15]. The human scalp SEP correlates of the secondary depolarization are not known.

Are these secondary components of medium-latency correlates of a secondary EPSP of the same neurons, which generate N20 and P25? They should increase with repetitive stimulation at intervals corresponding to a stimulation rate of 6–12/sec [11,12]. This would apply to N35 and N60, which in this study increased significantly with repetitive stimulation, while the primary components N20 and P25 were duly depressed (Figure 2 and 3). Depression of the primary response and enhancement of the secondary one by the second stimulus may be due to different mechanisms of origin. The second volley reaches the cortical neuron in a state of declining hyperpolarisation which suppresses the sodium current mediated primary response but creates favourable conditions for the activation of voltage dependent cation currents and low threshold calcium currents (It) underlying the secondary depolarisation [14,15]. On the other hand, reduction of IPSP efficacy with repeated stimulation might allow the emergence of an NMDA-receptor mediated late EPSP [16,17]. The marked difference of peak latencies of primary and secondary EPSP indicates polysynaptic generation of the latter. The late NMDA mediated EPSP may be nonetheless monosynaptic with a longer rise time. The expression of the NMDA-receptor of area 3b stellate cells may depend on the activity of intracortical connections from adjacent subareas of 3b, which represent neighbouring nerves.

P45 is another correlate of a primary EPSP, since it is depressed by repetitive stimulation. P45 is probably the human analogue of the SEP component P25 of the monkey. P25 was not associated with unitary activity in the monkeys primary somatosensory (S1) cortex [18]. The authors suggest, that this wave may reflect activity in area 5 or in the SII cortex. Loss of P45 after lesions of area 5 provides evidence in favour of this area. Area I, which generates the human P25, seem to lack a surface component of the augmenting response [13,19]. The surface-negative N60, which does not reverse polarity with precentral electrode location, shows an incremental response to repetitive stimulation, which is probably the correlate of a recruiting response. Surface negative recruiting responses are generated in cortical upper layers by repetitive stimulation of unspecific thalamic nuclei [20].

**Figure 4:**
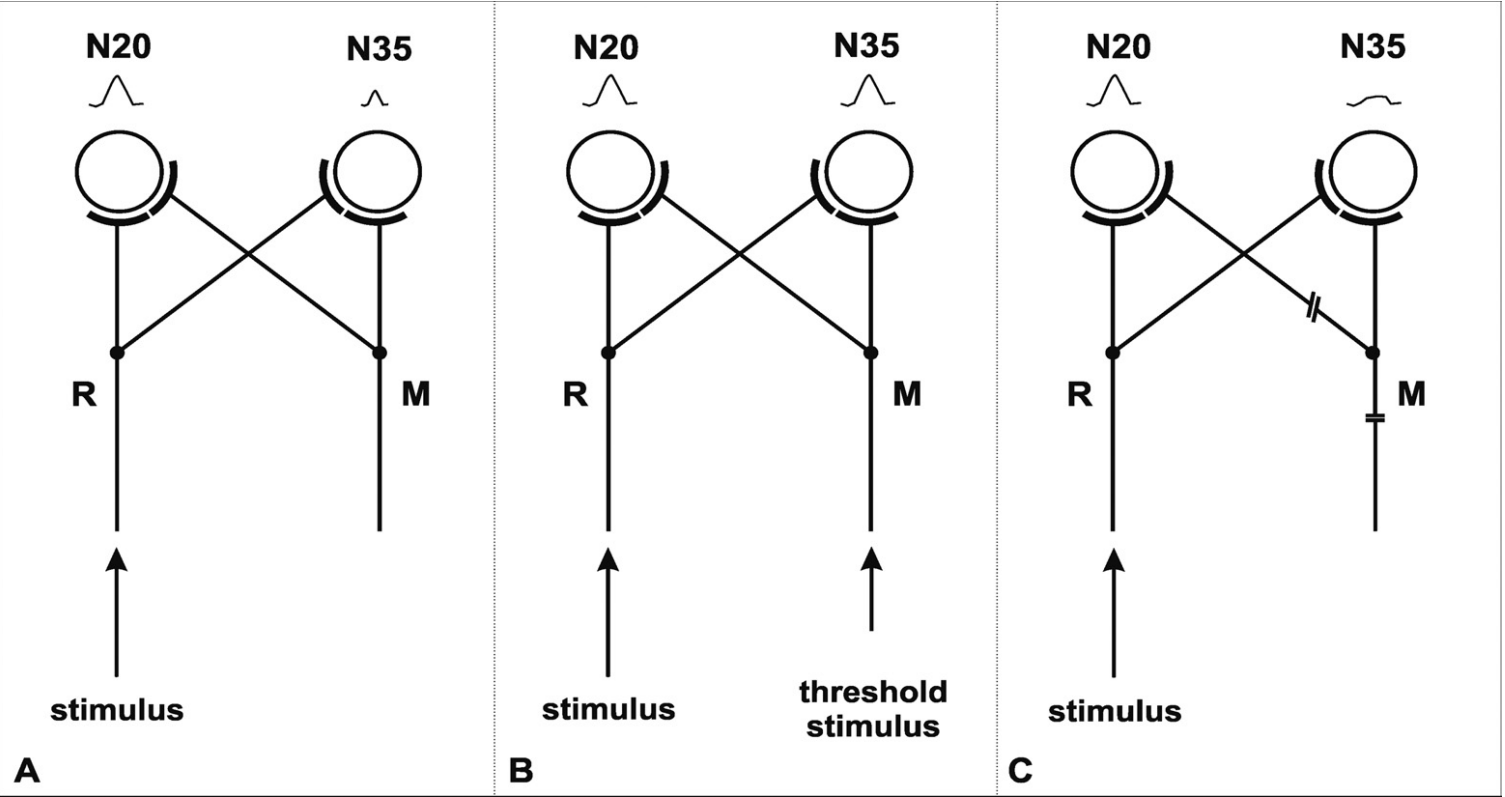
**Hypothetical explanation for changes of SEP-components**. Schematic illustration of SEP-component generation in area 3b with an intact (A, B) and a lesioned (C) neighbouring hand nerve (R = radial nerve afference; M = median nerve afference). **4A **The cortical recipients of radial nerve afference generate N20. Thalamo-cortical excitation spreads to the cortical representants of the neighbouring median nerve, which generate N35. **4B **Threshold co-activation of the median nerve enhances selectively N35. **4C **Inactivity due to nerve lesion makes the cortical representants of the lesioned nerve less excitable. Radial afference fails to co-activate cortical median neurons to generate N35.

To conclude, the cortical neuronal representants of innervation field borders of a sensory nerve seem to be co-innervated by afferents from the neighbouring nerve. The anatomical basis of this co-activation is the spread and overlap of axonal arborizations in the somatosensory cortex [21]. Co-activation may normally produce subliminal secondary depolarization in the middle layer of area 3b (N35), and in the upper layer of area 1 (N60) without spike discharge. Hypothetically, after nerve lesion the collateral innervation from the intact nerve may become unmasked and supraliminal and account for the expansion of the cortical projection area of the intact nerve as observed in the monkey [6] (Figure 4). In the human the influence of collateral innervation onto the cortical recipients of a lesioned nerve seems rather to decrease. The presumed SEP-correlates of co-activation (N35, N60) disappear. There seems to be no shift of the innervation field border of the intact nerve, since allaesthesia was never observed in our sample of 29 patients with hand nerve lesions.

## Conclusion

Secondary SEP-components of the excitatory response to nerve stimulation seem to be lost in cortical areas surrounding the denervated region. The presumed SEP-correlates of co-activation (N35, N60) disappear, suggesting that the influence of collateral innervation onto the cortical recipients of a lesioned nerve in humans seems rather to decrease.

## Competing interests

The author(s) declare that they have no competing interests.

## Authors' contributions

**RS **participated in the design of the study, the selection and examination of the patients, drafted the manuscript and the pictures/tables and performed the statistical analysis.

**UJ **participated in the examination of the patients and in the design of the study.

**MS **participated in the design of the study and in the statistical analysis and the drafting of the manuscript.
